# The therapeutic landscape of HIV-1 via genome editing

**DOI:** 10.1186/s12981-017-0157-8

**Published:** 2017-07-14

**Authors:** Alexander Kwarteng, Samuel Terkper Ahuno, Godwin Kwakye-Nuako

**Affiliations:** 1 Department of Biochemistry and Biotechnology, Kwame Nkrumah University of Science and Technology (KNUST), PMB, Kumasi, Ghana; 2 Kumasi Centre for Collaborative Research in Tropical Medicine (KCCR), Kumasi, Ghana; 3 Department of Biomedical Sciences, School of Allied Health Sciences, College of Health and Allied Sciences, University of Cape Coast, Cape Coast, Ghana

## Abstract

Current treatment for HIV-1 largely relies on chemotherapy through the administration of antiretroviral drugs. While the search for anti-HIV-1 vaccine remain elusive, the use of highly active antiretroviral therapies (HAART) have been far-reaching and has changed HIV-1 into a manageable chronic infection. There is compelling evidence, including several side-effects of ARTs, suggesting that eradication of HIV-1 cannot depend solely on antiretrovirals. Gene therapy, an expanding treatment strategy, using RNA interference (RNAi) and programmable nucleases such as meganuclease, zinc finger nuclease (ZFN), transcription activator-like effector nuclease (TALEN), and clustered regularly interspaced short palindromic repeats/CRISPR-associated proteins (CRISPR–Cas9) are transforming the therapeutic landscape of HIV-1. TALENS and ZFNS are structurally similar modular systems, which consist of a *FokI* endonuclease fused to custom-designed effector proteins but have been largely limited, particularly ZFNs, due to their complexity and cost of protein engineering. However, the newly developed CRISPR–Cas9 system, consists of a single guide RNA (sgRNA), which directs a Cas9 endonuclease to complementary target sites, and serves as a superior alternative to the previous protein-based systems. The techniques have been successfully applied to the development of better HIV-1 models, generation of protective mutations in endogenous/host cells, disruption of HIV-1 genomes and even reactivating latent viruses for better detection and clearance by host immune response. Here, we focus on gene editing-based HIV-1 treatment and research in addition to providing  perspectives for refining these techniques.

## Background

Human immunodeficiency virus-1 (HIV-1) infection is still a major contributor to global disease burden. The brunt of the infection is borne mostly by resource-limited populations [1]. Despite much effort by regional and international public health organizations, sub-Saharan Africa accounts for approximately 70% of all 36.73 million people living with HIV-1 world-wide [2]. On the other hand, the availability of early treatment therapies are changing the epidemiology of the disease, contributing to decreasing HIV-1 incidence as a result of drastic reductions of the risk of transmission of the infection [1].

One of the key challenges to the effective treatment and management of HIV-1 infection is the persistence of transcriptionally silent but replication competent integrated viral DNA (provirus) in long-lived memory CD4+ T cells, naïve CD4+ T cells, myeloid cells in the CNS, tissue-based macrophages and other sanctuary sites [3]. A larger proportion of latent HIV-1 is housed by resting CD4+ T cells in the periphery. Resting CD4+ T cells are less endowed with key transcriptional factors such as NF-kB, positive transcription elongation factor b (P-TEFb) and CDK11, all of which are important for HIV-1 replication [4, 5]. Ideally, one clinical important role of latent HIV-1 with regards to the pathogenesis of the disease is by functioning as a repertoire of the HIV-1 viruses for sustained infection, tropism or disease progression.

In many instances, latent viral reservoirs evade host immune response, therefore remain refractory to standard treatment strategies, such as antiretroviral therapies [6]. Several studies have reported viral recrudescence upon interruption or cessation of antiretroviral therapy. However, this scenario is correlated with increased risk of morbidity and definitely mortality among such patients with history of treatment interruption [7, 8].

The exact mechanisms mediating viral latency remains elusive. Previous studies propose that HIV-1 quiescence is predominantly driven by complex epigenetic mechanisms/pathways as well as transcriptional interferences by both viral and host factors [9]. Overcoming the barriers posed by latent HIV-1 will be key to the eradication of the infection. Several approaches have been proposed, some of which are under early stages of development, to target latent HIV-1. These strategies are predominantly based on the “shock and kill” strategy. The shock and kill strategy is a hypothetical term in which viral reservoirs are awaken, thereby making them susceptible to clearance by host immune defences and or therapeutic agents such as ARTs. Conversely, rather than awakening latent HIV-1 reservoirs, these viral reservoirs could be silenced by targeting key signaling pathways or molecules important for cytokine activation. Existing evidence shows that reduction of T cell activation is correlated with decrease HIV-1 associated inflammation in HIV-1+ individuals [10]. Moreover, murine studies using JAK and STAT inhibitors such as ruxolitinib and tofacitinib have demonstrated suppression of T cell activation. This suggests their high potential of being translated into clinical studies [11, 12].

Conversely, there are a number of pre-existing techniques/tools for “brute-force” activation of latent viral cells given that the chances of effectively clearing these cells are greatly increased by several folds after activation. Latent reversing agents (LRAs) such as histone deacetylase inhibitors (HDACis) promote acetylation and remodelling of the chromatin, therefore support enhanced expression of cell-associated HIV-1 RNA from latent viral reservoirs. However, there are a number of challenging results that report low coverage of all intended latent cells, thus only a small subset of latent cells were targeted by HDACi interventions [13–16]. This sheds lights on the complex signaling networks in vivo that are intricately fashioned to maintain memory cells (and for this matter HIV-1 infected memory cells) in a resting stage. The stochastic nature of latency reversal is a huge impediment to the study of LRAs activity over protracted periods and therefore supports the development of animal models, especially non-human primates, for in vivo studies [17].

Another class of LRAs, capable of reactivating HIV-1 in cell line models of latency are the BET bromodomain inhibitors (BETis) such as JQI [18]. Unfortunately, BETis are ineffective HIV-1 reactivating agents in human primary resting infected T cells primarily due to the inability of these humanized cells to express sufficient levels of essential transcription factors (TFs) [19, 20].

However, protein kinase C (PKC) agonists [21, 22] (such as prostratin, bryostatin and ingenols) [23–25] and MAPK agonists (including procyanidin) [26, 27] can reactivate HIV-1 in primary CD4+ T cells and almost all other cell line models. The success of PKC and MAPK agonists is based on their ability to increase cellular concentrations of required TFs needed for reactivation of primary CD4+ T cells in human cells [23]. The use of PKC and MAPK agonists are not without side effects. For instance, respiratory distress, muscle pain and toxicity even at therapeutic levels with prostratin and bryostatin use have been documented. Another cause for alarm, is the high cost of producing bryostatina, natural product derived from marine sources, warranting the need for the development of novel and cost-effective MAPK and PKC agonists such as synthetic bryostatin or igenol analogues with superior or comparable therapeutic efficacy with no or few side-effects [28, 29].

In contrast, HDACis and BETis mono-therapies have enormous therapeutic potential. Combined with other therapeutic agents, such as powerful immune stimulating agents, appear promising to effectively purge all latent viral reservoirs [29, 30]. Powerful immune activating agents, such as interleukin-2 (IL-2) [31] and anti-CD3 antibody [32] in combination with antiretroviral drugs has demonstrated enhanced HIV-1 expression from latent viral reservoirs and increased clearance of infected resting memory CD4+ T cells [33]. More specifically, LRAs in combination with powerful immune boosters may be used to induce viral proteins or processed antigens on cell surfaces that can be sufficiently recognized by the host immune cells such as cytotoxic T lymphocytes (CTLs) and NK cells for killing infected cells [34]. Similarly, activation of broadly neutralizing antibodies (bNAbs) with capabilities of recognizing several clades of HIV-1 together with even escape mutants represents another dimension being harnessed to kill HIV-1 latent/infected cells by host defences [35, 36]. Recently, chimeric antigen receptors (CARs), which can be engineered to recognize specific viral proteins have been developed to enhance T cell receptor avidity and activation. However, concerns over the longevity of CARs-bound cells as well as substantial off-target effects are limitations of the widespread application of CARs [37] for therapeutic purposes.

At a glance, the expanding promise of the various anti-HIV-1 “shock and kill” strategies leaves little room for reservations. Given that some latent HIV-1 activating agents are also capable of dampening CTL functions, there is the need for a more careful investigation into the mechanisms underlying these ‘shock” agents [37] in order to fine-tune these strategies where needed. Furthermore, continuous expansion of the already vast body of knowledge surrounding viral infection and molecular evolution is still needed. Studies relating to the regulatory mechanisms of HIV-1 gene expression during latency, in addition to the molecular underpinning underlying key viral processes such as HIV-1 nuclear mRNA export, splicing and translation will not only lead to more understanding of the virus but also promote the development of novel therapeutic strategies [3]. Moreover, a thorough understanding of the pathophysiology of infected cells at the various sanctuary sites, the effects of tissue microenvironment on viral latency, in addition to the canonical cell types involved in latency are urgently need to garner efforts towards targeting HIV-1 latent reservoirs. Taken together, it will be almost impossible to effectively purge all HIV-1 latent reservoirs without a deeper understanding of replication capacity and extent of viral expression at the various sanctuary sites [38].

## HIV-1 therapeutic landscape

The call to end the global pandemic might be achieved through two therapeutic approaches. Firstly, the sterilizing approach, which theoretically implies purging all HIV-1 latent reservoirs (as described above). Secondly, the functional cure approach, which seeks to empower the host immune defences to fight the infection and other opportunistic infections that arise as the disease progresses. Generally, both approaches are effective and heavily depend on HAART. Research into anti-HIV-1 gene therapy has intensified following the so called “Berlin-patient” where scientists eradicated HIV from his body after receiving a bone marrow transplant. Therefore, gene and nucleic acid based therapies including gene editing with programmable nucleases, RNA decoy, DNA/RNA aptamers, ribozymes, antisense, inhibitory proteins, fusion inhibitors and sh/siRNA have also been developed of which some are candidates for ongoing clinical trials (Table 1) [39–48].

**Table 1:** Anti-HIV-1 gene therapy landscape

Therapeutic approach	Gene therapy	Interventions	Targets	Identifier	Stage/status	Company/Institute
Drug shRNA peptide	Dual anti-HIV gene Transfer construct, LVsh5/C46 (Cal)	CCR5 shRNA; C46 peptide Busulfan	Host co-receptor; viral Env; transplant conditioning	NCT01734850	Phase I/II recruiting	Calimmune/CalTech/UCLA
shRNA	Long term follow up of delayed adverse events in Cal-1 recipients	Blood test for general health, complete blood count and Cal-1 specific analyses		NCT02390297	Recruiting by invitation	Calimmune/CalTech/UCLA
Endoribonuclease	Redirected MazF-CD4 autologous T-cells	CCR5 MazF	Host co-receptor	NCT01787994	Phase I ongoing	University of Pennsylvania
Ribozyme	Autologous CD34+ HSCs transduced with anti-HIV-1 ribozyme (OZ1)	Tat-vpr anti-HIV ribozyme	tat-vpr mRNA	NCT00074997	Phase II completed	Janssen-Cilag Pty Ltd, UCLA
Ribozyme	Long term follow up of OZ1 Gene therapy	Blood test for quantitative marking of the gene transfer product in PBMCs overtime		NCT01177059	Phase II Recruiting by invitation	Janssen-Cilag Pty Ltd, UCLA
Antisense	Tolerability and therapeutic effects of repeated doses of autologous T cells with VRX496	VRX496 antisense RNA	Env mRNA	NCT00295477	Phase I/II Ongoing/ not recruiting	University of Pennslyvernia/NIAID
Antisense	Safety and efficacy of T-cell genetic immunotherapy	VRX496 antisense RNA	Env mRNA	NCT00131560	Phase II Ongoing	VIRxSYS Corporation
Ribozyme	L-TR/Tat-neo in HIV+ Patients with Non-Hodgkin’s Lymphoma	Tat ribozyme	Tat-rev mRNA	NCT00002221	Phase II completed	Ribozyome/City of Hope
Inhibitory peptides	M87o autologous HSCs for HIV+ patients with malignant disease	C46 peptide	Viral Env	NCT00858793	Phase I/II suspended	University Medical Center Hamburg-Eppendorf
Ribozyme, inhibitory peptide	C46/CCR5/P140K modified autologous HSCs in patients with lymphoma	C46 peptide, CCR5 ribozyme, MGMTP140 K mutant	Viral Env, CCR5 mRNA, Alkylating agent resistance	NCT02343666	Phase I, recruiting	Fred Hutchinson Cancer Research Center/NCI/NHLBI
shRNA,peptide	Autologous transplantation of HSCs with LVsh5/C46 (Cal-1) for treatment of HIV-related lymphoma	CCR5 shRNA, C46 peptide	Host co-receptor, viral Env	NCT02378922	Phase I recruiting	Fred Hutchinson Cancer Research Center/NCI
shRNA, ribozyme,RNA decoy, drugs	rHIV7-shI-TAR-CCR5RZ-transduced HSC in patient with AIDS-related Non-Hodgkin Lymphoma	tat/rev shRNA, TAR decoy, CCR5 ribozyme, Prednisone, Rituximab, Etoposide, Doxorubicin Hydrochloride, Vincristine Sulfate, Cyclophosphamide	Viral mRNA, Viral tat proteinm, CCR5 mRNA, Transplant conditioning	NCT02337985	Pilot recruiting	City of Hope Medical Center/NCI
shRNA, ribozyme,RNA decoy, drugs	rHIV7-shI-TAR-CCR5RZ-transduced HSC in patient with AIDS-related Non-Hodgkin Lymphoma	tat/rev shRNA, TAR decoy, CCR5 ribozyme, Busulfan	Viral mRNA, Viral tat proteinm, CCR5 mRNA	NCT01961063	Pilot recruiting	City of Hope Medical Center/NCI
shRNA, ribozyme,RNA decoy, drugs	rHIV7-shI-TAR-CCR5RZ-transduced HSC in patient undergoing stem cell transplant for AIDS-related lymphoma	tat/rev shRNA, TAR decoy, CCR5 ribozyme, carmustine, cyclophosphamide, etoposide	viral mRNA, viral tat protein, CCR5 mRNA, transplant conditioning	NCT00569985	Pilot ongoing, not recruiting	City of Hope Medical Center/NCI
shRNA, RNA decoy	shRNA/TRIM5alpha/TAR decoy-transduced Autologous HSC in patient with HIV-Related Lymphoma	CCR5 shRNA, RNF88,TAR decoy	Host co-receptor, Gag p24, Viral tat protein	NCT02797470	Phase I/II	City of Hope Medical Center/NCI
RNA decoy	RNA decoy (ex vivo retroviral modified CD34+ HPC)	Rev reponse element decoy	Rev Protein	NCT00001535	Phase 0-pilot	Children’s Hospital
	Redirected high affinity gag-specific autologous T cells	WT-gag-TCR or alpha/6-gag-TCR	CD8 TCR	NCT00991224	Phase I completed	University of Pennslyvernia/Adaptimmune
ZFN	T-cells modified at CCR5 gene by ZFN SB-728mR	CCR5 ZFN	CCR5 DNA	NCT02388594	Phase 1 recruiting	University of Pennsylvania/NIAID
	Repeated doses of SB-728mR-T after cyclophosphamide conditioning in HIV+ on HAART	CCR5 ZFN (SB-728mR-T)	CCR5 DNA	NCT02225665	PHASE I/II ongoing	Sangamo biosciences
	Autologous T cells modified at CCR5 gene by ZFN SB-728	CCR5 ZFN	CCR5 DNA	NCT00842634	Phase 1 completed	Sangamo biosciences/ Uni. Of Pennsylvania
	Autologous T-cells genetically modified at the CCR5 gene by zinc finger nucleases (SB-728-T) in HIV-infected patients	CCR5 ZFN	CCR5 DNA	NCT01044654	Phase I completed	Sangamo Therapeutics
	Safety of ZFN CCR5-modified HPS/progenitor cells in HIV+	SB-728mR-HSPC infusion after busulfan conditioning	CCR5 DNA	NCT02500849	Phase 1	City of Hope Medical Center|Sangamo Therapeutics
	Autologous T-cells genetically modified at the CCR5 Gene by zinc finger nucleases in HIV-infected subjects	CCR5 ZFN (SB-728-T)	CCR5 DNA	NCT01252641	Phase I/II completed	Sangamo Therapeutics

## Chemotherapy (drugs)

Treatment with HAART, the primary treatment strategy, has greatly impacted the epidemiology of HIV-1 infection changing the previously life-threatening disease into a chronic disease. The move by world leaders to make these drugs available to endemic regions, particularly developing nations, led to significant reduction in the number of AIDS-related deaths as well as an increase in the quality of life of infected individuals [49, 50]. However, HAART is intensive and life-long, usually leading to treatment fatigue, with considerable side effects [50]. Moreover, poor pharmacokinetics of these drugs and tissue toxicity, on top of viral resistance after prolonged treatment, have also been widely documented throughout the volumes of scientific literature and clinical practice [51]. Added to these are the huge economic and logistical challenges borne by developing countries in order to make treatment sustainable. The constellation of drawbacks warrants the development of robust and effective treatment regimens to supplant HAART, which will result in better treatment and management as well as the possible eradication of the virus. The advancement of biomedical research and engineering, nano-delivery of drugs to specific and key anatomical barriers hold the promise of increasing the efficiency of HIV-1 chemotherapy [52–54].

## Nano-medicine

Anti-HIV-1 nanomedicine involves the administration of minute (on the nano-scale, 10^−9^ m) anti-HIV-1 therapeutic agents to allow precise delivery to virtually any therapeutic target sites particularly HIV-1 sanctuary sites such as the central nervous system. The development of biocompatible, biodegradable, non-toxic nanoparticles are feasible depending on the material of manufacturing and are therefore a keen research focus in biomedical engineering [55]. Nanotechnology-based anti-HIV-1 therapeutic agents could range from various drug formulations to gene therapy toolkits (such as RNA interference and anti-HIV-1 ribozymes), which could either be bound to or encapsulated in nano-carriers [56]. Anti-HIV-1 nano-based therapeutic agents have been upheld for their ability to facilitate stable and prolonged drug circulation coupled with the ability to specifically target intended cells/tissues with improved toxicity profiles and low side effects [53]. These ground-breaking techniques facilitate the permeation of the blood–brain barrier of the CNS with remarkable precision and accuracy [57]. However, a move to translate nanotechnology-based anti-HIV-1 agents into clinical practice would require a critical review of existing delivery routes as well as the development of novel delivery routes for nano-formulations. This precaution has now become necessary given that both the pharmacodynamics and pharmacokinetics (absorption, distribution, metabolism and elimination) of nano-formulations are at times affected by the mode of delivery [58]. Such assessments would deepen our knowledge of the efficacy and safety of this treatment approach while opening up new frontiers for HIV-1 research and treatment. A critical assessment of the cost-to-benefit ratio is of equal importance, particularly to ensure wider coverage of middle and lower income individuals as well as resource-limited countries in the upcoming years.

## Anti-HIV-1 RNA interference (RNAi)-based therapeutic landscape

RNA interference is a widely used technique in endogenous cells and biomedical research for regulating gene expression and cellular defense against viral infections [59]. The technique has served as the bedrock for the elucidation of complex signaling pathways in biological systems, functional genomics and gene therapy. RNA interference has been extensively applied to the elucidation of HIV-1 pathogenesis as well as identification of novel therapeutic targets for controlling HIV-1 infections.

microRNAs (miRNAs) and small interference or short hairpin RNAi (si/shRNA) are the two RNAi toolkits used for RNAi mediated gene silencing studies. miRNAs are short endogenous single-stranded oligonucleotides (~22 bp) that specifically binds to and suppresses mRNA expression. They are therefore capable of modulating key cellular processes, such as developmental processes and even influencing the pathogenesis of diseases [60]. In contrast, sh/siRNAs are double stranded RNA molecules (21–23 bp) generated by the cleavage of either an endogenous or exogenous RNA. Dicer is a ribonuclease, which functions primarily by cleaving RNA to generate sh/siRNAs for targeted gene silencing. Following cleavage, siRNA forms a large complex, RNA-induced silencing complex, (RISC) with helicase (in the case of plants and drosophila cells) and other nucleases such as Argonaute (Argo) proteins. The complex facilitates uncoiling of the siRNA thus promoting precise RNA binding to targeted transcripts, which could lead to sequence-directed gene silencing by mRNA repression [61], translational repression [62], or heterochromatin formation [63]. In humans, the transactivation response RNA-binding proteins (TRBP) is an addition to the RNA-induced silencing complex.

There are two main pathways for targeted gene silencing by RNAi, namely, post transcriptional gene silencing (PTGS) [64] and transcriptional gene silencing (TGS) [65]. PTGS occurs mostly in the cytoplasm where complementary base-pairing of siRNA with target mRNA leads to the degradation of the targeted mRNA. TGS occurs predominantly in the nucleus but unlike PTGS, the target sequences for TGS are usually gene promoter sequences. Therefore, binding of the siRNA to these sequences induces epigenetic silencing of the targeted gene. Although, TGS has been least explored for gene silencing by RNAi, both pathways have been the heart for the development of powerful RNAi therapeutic agents [66, 67]. RNAi can be achieved by targeting conserved viral mRNAs, particularly the ones involved in viral entry, reverse transcription and integration as well as genes that encode structural and enzymatic proteins needed for viral assembly and infection (Table 2). In principle, cutting or editing small pieces of nucleotides in the coding region may give rise to critical mutations that will disrupt the activities or viability of the virus [68]. Host cellular co-factors such as the CD4, CCR5, and CXCR4 receptors essential for viral infection have thus been the center of RNAi based HIV-1 therapy. Moreover, other host co-factors such chaperonin, LEDGF/p75, and importin-7 involved in HIV-1 integration are veritable RNAi as well as other gene therapy targets for anti-HIV-1 treatment.

**Table 2:** Potential gene therapy targets for HIV-1 therapy

HIV-1 gene targets	Function	Reference
Gag	Proteolytic processing of the HIV-1 genome	[135, 136]
Pol	Transcription	[137]
env	Receptor binding and fusion	[138, 139]
tat	Transcription or RNAi modulation	[140]
rev	Reverse transcription, integration	[141]
nef	Immune modulation	[124, 142–145]
pol (integrase)	Integration	[146, 147]
pol (reverse transcriptase)	Reverse transcription	[148]
Promoter	Transcription	[149]
Long terminal repeats	Genome expression	[145, 150]
P17	Assembly and budding of HIV-1	[68]
Cellular targets		
CCR5	Receptor binding and fusion	[151]
CD4	Receptor binding and fusion	
CXCR4	Receptor binding and fusion	[152, 153]
Chaperonin	Integration	[154]
LEDGF/p75	Integration	[155]
Importin-7	Integration	[156]
Cyclin T1	Transcription	[157]
P-TEFb	Transcription	[158]
Tat-SF1	Transcription	[159]
SPT5	Transcription	[160]
DDX3	Export	[161]
SOCS1	Trafficking or immune modulation	[162]
TRBP	Immune modulation or RNAi pathway	[163, 164]
TNPO3	Nuclear entry of viral pre-integration complex	[141, 165]

## Challenges with RNAi

Conversely, despite the popularity of RNAi mediated anti-HIV-1 treatment and research, some trade-offs exist. Of particular importance, is the high likelihood of generating viral escape mutants also known as siRNA escape mutants. This is due to the high error rate of the viral reverse transcriptase (1 in 1000 nucleotides per replication cycle) hence a change in even 1 bp could lead to mutations in the targeted sequences therefore limiting the regulatory effects of siRNAs [69]. However, one of the surest ways of offsetting this challenge is by deploying multiple anti-HIV-1 siRNAs. Another is the combination of other anti-HIV-1 therapeutics, in particular antiretroviral drugs.

Some inherent factors of siRNA, such as anionic charge and large molecular weight, makes it difficult for successful migration across the cell membrane by simple diffusion, thereby preventing its full utilization in certain jurisdictions [56]. In addition, siRNAs are susceptible to degradation by RNAses making it imperative for the development of novel delivery methods. RNA nanotechnology and hydrodynamic cell transfection are making it more practical to delivery RNAi toolkits to targeted cells for research and therapeutic purposes. Furthermore, plasmids and lentiviral vectors that encode siRNA have been useful in delivering RNAi therapeutic agents to targeted cells or tissues for gene therapy.

## Anti-HIV-1 gene editing therapeutic landscape

The arrival of gene editing tools such as zinc finger nucleases (ZFNs), transcription activator-like effector nucleases (TALENs), and clustered regularly interspaced short palindromic repeats/CRISPR-associated proteins (CRISPR–Cas9) has revolutionized biomedical research. Similar to other seminal scientific breakthroughs, the adoption of these reagents by modern research have increased exponentially since their invention—and undoubtedly proves promising as arsenals for eradicating HIV-1 infections as well as other viral infections (such as Hepatitis B and C virus, human papilloma virus and herpes simplex virus) even in resource-limited populations.

Previous and ongoing applications of these proof-of-concept technologies may not be limited to programming cells to be permanently resistant to HIV-1 infection and interfering with HIV-1 replication by targeting viral proteins such as Tat, Env, and Gag. HIV-1 resistant cells can be generated by editing genes that encode important cellular factors needed for viral invasion such as CCR5 or CXCR4 co-receptor (Table 2). Ideally, conferring resistance to HIV-1 susceptible or infected cells would require highly efficient, precise, accurate in vivo strategies. However, in vivo delivery approaches are imperfect and cannot produce precise, distributed and sustained delivery of therapeutic agents to targeted cells. Current delivery methods rely on ex vivo manipulation of autologous cells reintroduced into the host after treatment to observe the intended therapeutic effect.

## Zinc-finger nucleases (ZFNs)

**Fig. 1:**
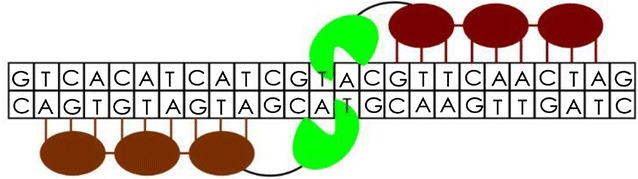
Zinc finger nucleases

The structure of ZFNs is basically the combination of 2 domains: the nonspecific *FokI* restriction endonuclease for cleavage of targeted sequences and the custom-designed Cys_2_-His_2_ zinc-finger proteins (ZFPs) for specific DNA-binding (Fig. 1). The domains are stabilized by zinc ions. Unlike other DNA binding proteins, which depends on the twofold symmetry of the double helix, ZFNs have the advantage of being linked linearly in tandem to recognize nucleic acids of varying lengths, allowing unprecedented combinatorial possibilities for specific gene targeting and manipulations [70]. Ideally, ZFN subunits recognize target sequences in a head-to-tail conformation. After recognition and binding of ZFNs to specific genomic loci, there is the dimerization of the two nuclease domains which leads to a double-stranded break (DSB) of the targeted DNA [70].

ZFNs have been widely used as intervention in several gene therapy clinical trials. Out of the six ZFN anti-HIV-1 clinical trials, three studies have been completed with promising results (NCT00842634, NCT01044654, and NCT01252641). The studies involved the removal of white blood cells that contain CD4+ T cells from consented HIV-1+ patients. The extracted cells were genetically modified by ZFNs, which lead to mutations in the CCR5 gene encoding proteins that function as co-receptors on the surfaces of CD4+ T cells needed for HIV-1 entry. The genetically modified cells were then re-infused back into the individuals with the expectations that these new cells will remain resistant to HIV-1 and possibly produce several generations of HIV-1 resistant cells eventually. Furthermore, another ongoing clinical trial (NCT02500849) that is being sponsored by Sangamo Therapeutics and the City of Hope Medical Center applies CCR5-targeted ZFNs to Hematopoietic Stem/Progenitor cells in HIV-1 infected patients. One of the primary outcomes of the study was to evaluate the safety of SB-728-mR-HSPC (CCR5-targeted HSPCs using ZFNs) after bulsulfan dose in HIV-1 infected individuals. Indeed, this particular study is timely due to the associated risk of developing tumours when HSPCs are genetically manipulated [71].

On the other hand, there are credible reasons to explore other gene editing techniques other than ZFNs. Despite being the forerunner of all gene editing techniques, challenges with engineering customized proteins needed for precise DNA-protein interaction by ZFN are hampering further exploitation [72]. In addition, the development of off-target effects are anecdotes, which needs careful consideration when using ZFNs.

## Transcription activator-like effector nucleases (TALENs)

**Fig. 2:**
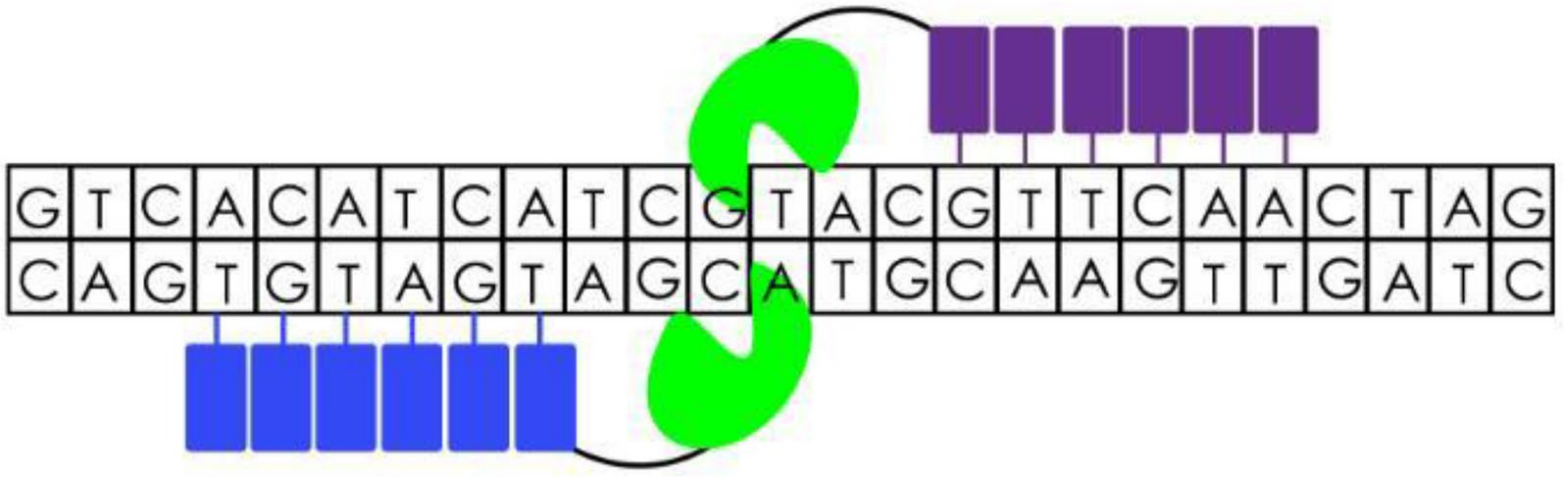
TALENS

TALENs are structurally similar to ZFNs comprising of a TALE DNA-binding region and a *FokI* restriction endonuclease domain (Fig. 2) [73]. Transcription activator-like effectors (TALEs) are naturally occurring DNA binding proteins from the plant bacterial pathogen, Xanthomonas [74]. In contrast to ZFNs where each finger module recognizes three target DNA nucleotides, TALE proteins contain a highly conserved, central domain, usually consisting of 33–35 amino acid TALE repeats for which each protein monomer is capable of recognizing single base pairs of the target DNA [75]. However, the specificity of these DNA-protein interactions are dictated by two hypervariable residues as shown by studies, which investigated the crystal structure of TALEs bound to DNA. The results from these studies show that each TALEN repeat forms a two-helix structure connected by a loop which presents the hypervariable residues into the major groove as the protein wraps around the DNA in a super-helical structure [76, 77]. The flexibility of joining these modular TALE repeats to form long arrays with custom DNA-binding specificities has proved useful in targeted gene editing of a variety of cells for therapeutic purposes [78–80]. Although TALENs are cost effective when compared to ZFNs, they are difficult to generate. The bulkiness of both ZFNs and TALENs makes it more difficult to deliver these reagents to several targeted cell types [81]. It has been shown that the presence of multiple sequence repeats in TALEN genes renders them unsuitable cargos for lentiviral vector repeats [81]. However, the single-nucleotide precision give rise to superior editing efficiency with minimal off-target and cytotoxicity effects when compared to ZFNs thereby making TALENs good candidates for sequence-specific genome modification [82].

TALENs have not yet been applied for HIV-1 treatment in clinical studies. However, several experimental studies have shown promising results with potential for optimization for large scale anti-HIV-1 treatment. Shi and colleagues recently generated 28 CCR5-TALEN screens to target several domains on the CCR5 of CD4+ T cells [82]. CCR5-ZFNs similar to the anti-HIV-1 ZFNs currently being applied for ongoing clinical trials were used as controls in this study for comparative studies. The results were remarkable and showed increased editing efficiency and minimal cytotoxicity activity compared to the CCR5-ZFN currently undergoing clinical trials [83]. Furthermore, cleavage of HIV-1 matrix protein P17 gene sequences with custom designed TALENs delivered by lentiviral vectors to Jurkat-HIV-1 cell lines has been reported with editing efficiency of ~43% [68]. HIV-1 P17 helps in the assembly and budding of HIV-1, and shows relatively little sequence diversity, therefore serves as a suitable candidate for clinical applications. TALENS have been used to target a highly conserved sequence in the transcription response element of the HIV-1 proviral DNA [84]. The HIV-1 TALEN constructs used were able to achieve editing efficiencies ranging between 30 and 60%. Elsewhere capsid modified adenovirus (helper dependent adenovirus, HD-Ad5/35) vectors for ZFN and TALEN-mediated CCR5 truncation of human CD34+ hematopoietic stem cells (HSCs) has been documented [85]. HD-Ad5/35 vectors target the CD46 receptor constitutively expressed on all HSCs [86] and are effective transducers of human CD34+ HSCs in vitro with minimal effects of cytotoxicity [87]. HD-Ad5/35 have a large carrying capacity with capabilities to efficiently transduce primitive subsets of HSCs [88]. Moreover, Ru and colleagues [89] used a cell-penetrating peptide (TAT peptide, YGRKKRRQRRR) bound to ZFN and TALEN to disrupt genes encoding the CCR5 co-receptors of human induced pluripotent stem cells (hiPSCs). Cell-penetrating peptides (CPPs) such as TAT, are promising therapeutic delivery systems that have been used for the treatment of several diseases [90]. Their versatility is based on the fact that CPPs are applicable to all cell types with high transduction efficiency under controlled administration [91]. CPPs have been used for delivery of large cargos including biomolecules (such as nucleic acids and large proteins with low transmembrane permeability) [91–94] and drugs [95, 96]. A dose-dependent TAT-TALEN activity was observed under hypothermic conditions with disruption efficiency of 3 and 5% with HeLa and hiPSCs respectively. There was a lack of certainty of the factors that contributed to the differences in the editing efficiencies in the different cell types. Perhaps the differences in cell membrane composition and endocytosis capabilities were contributing factors that affected the varying CCR5-edting efficiency of HeLa and hiPSCs. The limited use of TAT-ZFN is due to its low protein expression and low binding affinity which largely contributed to challenges in purifying TAT-ZFN proteins for analysis in cell culture. Interestingly, an earlier study has reported considerable success with TAT-ZFNs for cell transduction and gene editing [97]. Additional studies are required in light of these contrasting results to support the conclusion that TAT-TALENs are superior to TAT-ZFNs for anti-HIV-1 therapeutic purposes.

On anti-HIV-1 experimental studies with huge therapeutic potentials, another study reported high-rate CCR5 knockout (>90% in PM1 and >50% in primary T cells) with relatively low off-target activity using CCR5-Uco-TALENs delivered into T cells by mRNA electroporation [98]. Conversely, Wang et al. [99] attained an editing efficiency of 40% of TRIM5α (tripartite motif containing 5) genes of rhesus macaques using TALENs. TRIM5α, among other HIV-1 capsid-binding proteins such as Fv1 and TRIMCypA are well characterized anti-HIV-1 proteins, which restricts early HIV-1 replication in non-human primate cells [100]. TRIM5α consists of RING, B-box 2, coiled-coil and B30.2 (SPRY), the major determinant of anti-HIV-1 potency [101, 102]. Although the human orthologues (TRIM5αhu) does not confer significant viral resistance, point mutations in the capsid-binding domain of human TRIM5αhu shows high anti-HIV-1 activity [103]. The exploitation of TRIM5α transgenes as candidates for anti-HIV-1 gene therapy is currently ongoing [104, 105]. The emergence of escape variants due to the fast evolution rate of HIV-1 are major potential challenges that may hamper the development and scalability of this therapeutic strategy.

Conversely, the lens epithelium-derived growth factor (LEDGF/p75) is a cellular co-factor needed for tethering and proper integration of HIV-1 genome into the host genome and thus remains an attractive therapeutic target. Gene therapy techniques such as RNAi and ribozymes have shown minimal success in knocking-out *PSIP1*, the gene which encodes LEDGF/p75, needed to confer HIV-1 resistance to cells. This is due to the fact that even a small residual of the tightly chromatin-bound protein is just enough to promote integration function [106]. Recently, Fadel and colleagues [107] provided the proof-of-concept by demonstrating that TALENs could effectively knockout *PSIP1* genes thereby blocking HIV-1 propagation in human cell lines.

Taken together, although there are currently no clinical studies applying TALENs as interventions for HIV-1 treatment, the existing pool of evidence from experimental studies indicates that TALENs are potential candidates for future anti-HIV-1 therapeutic agents.

## Clustered regularly interspaced short palindromic repeats/CRISPR-associated proteins (CRISPR–Cas9)

**Fig. 3:**
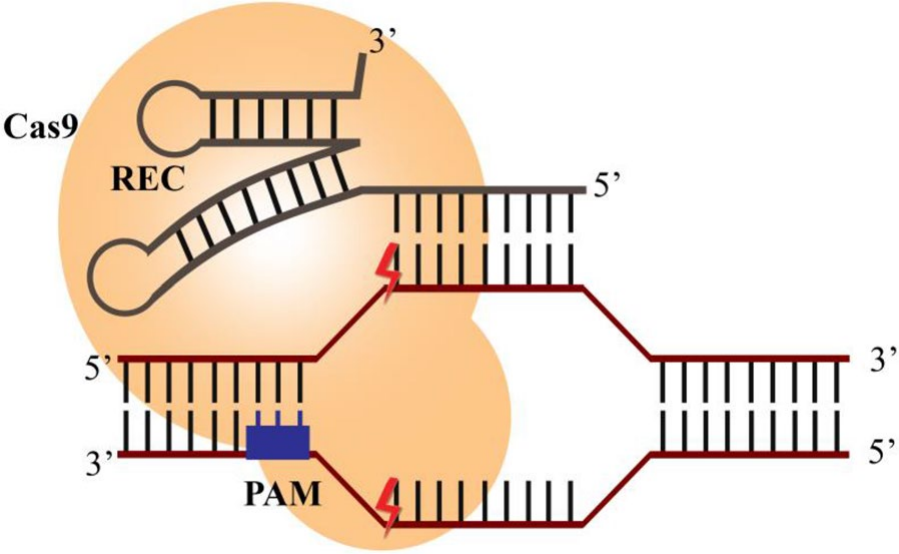
CRISPR–Cas9

**Fig. 4:**
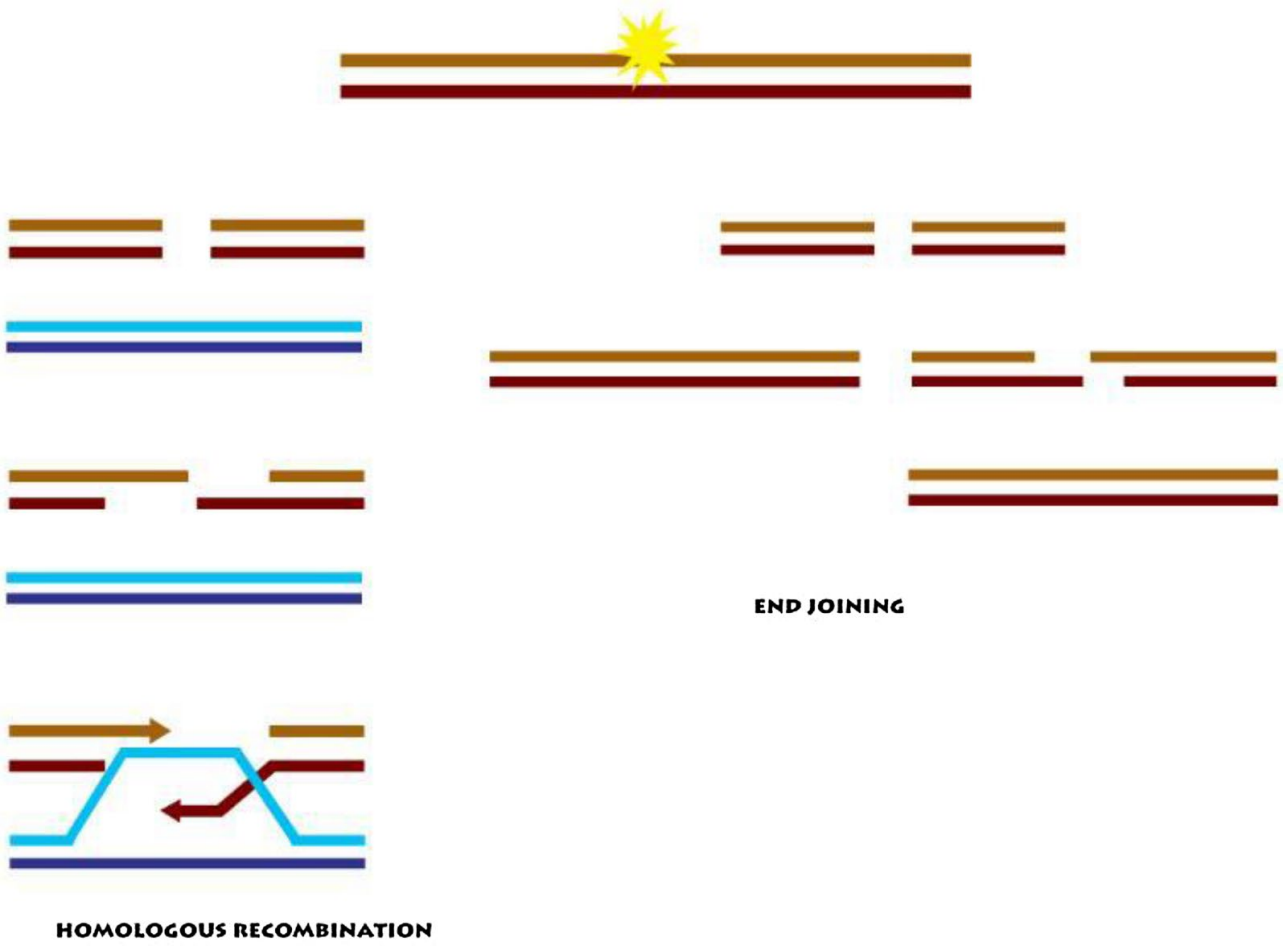
DNA repair mechanism

The emergence of CRISPR–Cas9, a proof-of-principle technique based on the adaptive immune systems of bacteria and archae [108] has transformed the therapeutic landscape of HIV-1. CRISPR–Cas9, has gained so much popularity in the research community due to the preciseness, cost-effectiveness and simplicity with its design thus allowing superior genetic manipulations of targeted sequences [109]. Unlike the former designer nucleases (ZFN and TALENs) CRISPR–Cas9 uses a specially designed guide RNA (gRNA) to direct a nuclease (Cas9) to specific genomic loci for genomic modification (Fig. 3) [110]. The disrupted genomic DNA is then repaired either by non-homologous end joining (NHEJ) or homologous recombination (HR) (Fig. 4). DNA repair via non-homologous end joining usually leads to mutations that interrupt the open reading frame, which could lead to gene inactivation when a template is provided.

Currently there are no ongoing clinical trials using CRISPR–Cas9 as anti HIV-1 therapeutic intervention. This is not surprising given the labyrinthine process and regulatory network surrounding approval for running a clinical trial. However, preclinical studies have demonstrated the importance of this approach as a golden tool for anti-HIV-1 therapeutic intervention. For example, Wang et al, used lentivirus vectors to express CCR5-sgRNA and Cas9 to knockout CCR5 co-receptors of CD4+ T cells thereby conferring HIV-1 resistance to these cells [111]. In addition, no mutations were reported at potential off-target sites that were homologous to the CCR5-sgRNA, even several days (85 days) after transduction. Furthermore, CRISPR–Cas9 system was recently used to remove the entire HIV-1 genome, spanning the full length 5′ and 3′ LTRs, of integrated HIV-1 proviral DNA from latently infected human CD4+ T-cells with no off-target effects [112]. The CRISPR–Cas9 mediated proviral DNA excision had no significant deleterious effect on several cell health indices such as cell viability, cell cycle progression and apoptosis. Moreover, continuous expression of sgRNA and Cas9 nuclease by T cells where HIV-1 was eliminated, showed protection from new HIV-1 infection as compared to T-cells expressing Cas9 or sgRNA alone. CRISPR–Cas9 has also been successfully used to activate latently infected cells to promote better detection and clearance of these latent cells by effector immune cells [109].

## *Natronobacterium gregoryi Argonaute* (NgAgo): the next golden editor?

Another valuable addition to the gene editing toolbox has been NgAgo, a DNA-guided endonuclease [113] with possibilities of generating site-specific modification of human cells. Unlike CRISPR–Cas9, NgAgo-gDNA system operates without a protospacer-adjacent motif (PAM) with remarkable low tolerance to guide-target mismatches as well as high efficiency in editing (G+C)-rich genomic loci. Although promising, the reproducibility of the original protocols could prolong the huge benefits such a tool could offer biomedical researchers with profound interest in gene modifications [114]. There are no reported studies using this latest addition to the genome editing toolbox for HIV-1 treatment and research, suggesting the need for future research to explore the potentials of NgAgo for HIV-1 research.

### Challenges and perspective

Programmable nucleases could be exploited to deepen current knowledge of host-viral interaction and to create improved animal models that mimics HIV-1 infections in humans. As a result our current knowledge of the molecular pathogenesis of the infection would be enhanced. However, genetic manipulations using non-human primates can be problematic due to several factors such as slow sexual maturity, possibility of generating mosaic mutations, off-targets, and low quality of publicly available non-human primate gene sequences, in particular monkey, as well as expensive embryos [72, 115]. The arrival of CRISPR–Cas9 [116] and TALENs [117] are breakthroughs of the century, which have facilitated the successful generation of transgenic HIV-1 non-human primate models to elucidate the key components in HIV-1 susceptibility, infection and immunobiology. Furthermore, programmable nucleases could boost studies with particular focus on the interplay between the adaptive immune system and HIV-1 in mice models after successful substitution and expression of murine genes with human homolog (such as cytokines) [72].

HIV-1 researchers have battled with some crucial challenges while experimenting with recent designer nucleases. These include editing inefficiency, imperfect delivery systems, off-targets editing and cytotoxicity in addition to immune intolerance [118].

The development of novel delivery systems that can target precisely intended cells with little possibility of not being tolerated by immune defences are crucial in moving forward.

**Fig. 5:**
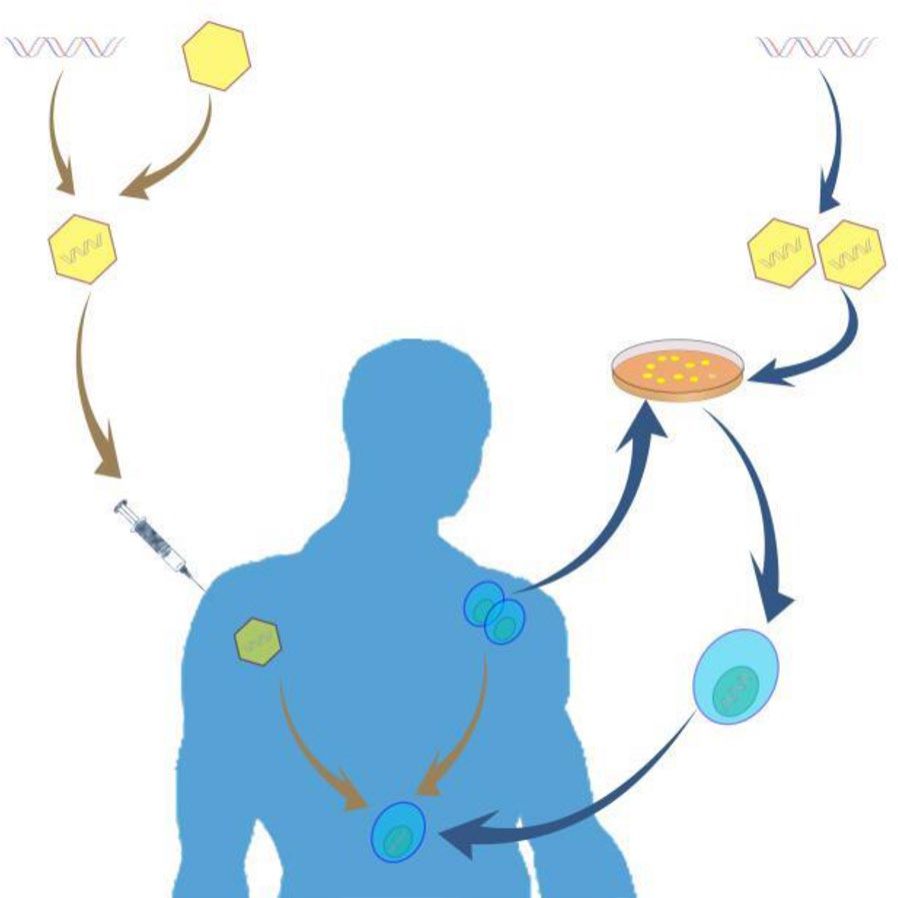
In vivo and ex vivo delivery methods

Gene therapy interventions can be achieved either by in vivo or ex vivo methods. In vivo delivery approaches relies on packaging into nanoparticles and administering directly to the patient through intravenous infusions. Ex vivo approaches involve sampling of host cells, culturing and treatment of target cells, such as CD4+ T-cells or CD34+ HSCs with biological interventions after which treated cells are infused back into the patient (Fig. 5). Transduction of targeted cells could be achieved either by nanodelivery or with biological vectors, such as adenovirus and lentivirus vectors. Adenovirus and lentivirus vectors are the two main biological delivery systems for targeted gene editing by programmable nucleases. However, the two viral vectors demonstrate differential integrity profiles. Adenovirus vectors are superior in transducing targeted cells with programmable nucleases and promote high site-specific double-stranded DNA breaks [119]. Scaling-up production and processing for large scale anti-HIV-1 gene therapy needs further investigation.

Until recently, the detailed mechanisms of viral escape (or resistance) following treatment with anti-retrovirals, small molecule inhibitors, and gene editors such as CRISPR/Ca9 were enigmatic. In the case of gene-editing antiviral therapies, resistance could result due to the introduction of target site mutations that would usually permit viral replication but inhibit endonuclease binding and cleavage [120]. Another cause for viral resistance is when a defective viral gene target is restored to its wild-type state by a recombination event with a functional viral genome. A study by De Silva Feelixge et al. [121] supported existing theories of HIV-1 resistance by demonstrating that insertional mutations introduced into the HIV-1 provirus following ZFN therapy enabled virus replication and ZFN cleavage resistance. The study showed similar replication levels of mutant and wild-type viruses and the persistence of mutant progeny even in activated primary T cells. Their findings stressed the need for combination therapy that would target multiple regions of the viral genome to avert the emergence of viral resistance.

Similarly, recent studies have supported previous theories of the mechanisms of HIV-1 resistance following treatment with CRISPR–Cas9 [122, 123]. The former used HIV-1 evolution experiments with CRISPR–Cas9, and revealed rapid and consistent viral escape even when conserved HIV-1 sequences were attacked by CRISPR–Cas9 [123]. This was done by sequencing the entire HIV-1 escape variants, which provided evidences of nucleotide insertions, deletions, and substitutions around the Cas9/gRNA cleavage site as a result of an imperfect non-homologous end-joining pathway of DNA repair pathway [123]. Conversely, the latter study supported these findings by showing mutations at HIV-1 pol, env, and LTR target sites which contributed to viral resistance [122].

Insight into the detailed mechanisms of Cas9/sgRNA HIV-1 resistance would be invaluable to the development of novel strategies to mitigate several viral escape. Nevertheless, some precautionary measures could be put in place to avoid incidence of escape mutants. CRISPR–Cas9 multiplex system could be fully exploited using multiple sgRNA to target conserved regions of the viral genomes [124]. Alternatively, customised or modified Cas9 proteins (such as Cpf1) [125, 126] or SpCas9 with novel PAM specificities [127] could be programmed to target sequences outside the targeted regions. This strategy would ensure that mutations arising from NHEJ repair will not prevent Cas9/sgRNA binding and DNA cleavage which previously were the cause of viral resistance.

Despite the discovery of smaller, yet effective Cas9 nucleases and improved strategies for transducing both CD4+ T cells and CD34 hematopoietic stem cells via lentiviral vectors [128, 129], there are substantial challenges with in vivo delivery of CRISPR–Cas9-encoding genes to certain cell types. Of most importance is the risk of insertional oncogenesis associated with integrating lentiviral vectors for successful gene transfer into cells and tissues. However, the use of highly efficient virus-like particles (such as the newly developed Lent-One Trans Vectors) that effectively packages the transcription activator joined with the Cas9 and single guide RNA, (Vpr-Cas9/sgRNA) are recommended for transient delivery into target cells. This could save HIV-1 researchers who are often trapped in the limbo of finding suitable delivery systems for CRSIPR-Cas9 [130].

In an attempt to combat insertional mutagenesis at antiviral therapeutic sites, the use of small molecule inhibitors [131], RNAi-based suppressors [132] and other chemotherapeutic agents, [133, 134] which silence specific enzymes involved in the NHEJ pathway while promoting homology-directed repair (HDR) could be adopted. Additionally, given the drawbacks of HAART as well as the limitation of current genome editing tools, exploring the synergistic power of combinatorial therapy (such as CRISPR–Cas9 with HAART) in actively suppressing HIV-1 replication could advance current efforts in finding a functional cure to HIV-1 in resource-limited countries.

## Conclusion

In this review, we have highlighted some of the mechanisms underlying the application of genome editing as a major control to augment existing strategies against HIV-1 infection. It will be interesting to investigate the efficacy of clearing HIV-1 proviral DNA while shedding more light on the outcome of such strategies on the health and safety of individuals.

While the research community is still keen on finding an anti-HIV-1 vaccine, gene therapy a budding therapeutic approach has seen many developments with promising potential for perpetually eliminating the disease.

Single gRNA has been shown to mediate suppression of HIV-1 replication following treatment. In addition to T cells we anticipate that genome editing approaches could be further explored to target other immune cell populations such as monocytes, macrophages and dendritic cells to harness functional treatment of HIV-1 infections. While most research focus on either the application of CRISPR–Cas9 in vitro or in vivo, there is the need for novel genome editing protocols, which are capable of transmigrating the blood-brain barrier (BBB) to target HIV-1-infected and latently-infected HIV-1 brain reservoirs to overcome the incidence of neuro-acquired immunodeficiency syndrome (neuroAIDS) in the brain [57]. Despite the fast pace of current research to end HIV-1, there is compelling evidence, as highlighted throughout this communication, that fighting HIV-1 cannot be achieved using a single therapeutic strategy but by a combinational approach of existing and new strategies yet to be developed. Given that the brunt of these infections are borne largely by developing countries, such as sub-Saharan Africa there is the need for careful consideration of the scalability and cost of promising gene therapy in order to make such treatments deliverable to the world’s bottom billion. Nevertheless, given the current hurdles with CRISPR–Cas9 and other recent genome editors, it is imperative for intensive and collaborative research across various biomedical disciplines to develop better strategies for optimization of these tools while opening up new avenues for the treatment of HIV-1.

## Abbreviations

CRISPR–Cas9: clustered regularly interspaced short palindromic repeats/CRISPR-associated protein 9
HAART: highly active antiretroviral therapy
HDR: homology-directed repair
NHEJ: non-homologous end joining
NgAgo: *Natronobacterium gregoryi Argonaute*
sgRNA: single guide RNA
TALENS: transcription activator-like effector nucleases
ZFNS: zinc finger nucleases
